# Monitoring secondhand tobacco smoke remotely in real-time: A simple low-cost approach

**DOI:** 10.18332/tid/104577

**Published:** 2019-03-05

**Authors:** Ruaraidh Dobson, Laura J. Rosen, Sean Semple

**Affiliations:** 1 Institute for Social Marketing, University of Stirling, Scotland, United Kingdom; 2 Sackler Faculty of Medicine, Tel Aviv University, Tel Aviv, Israel

**Keywords:** monitoring, environmental tobacco smoke, indoor air quality, secondhand smoke

## Abstract

**INTRODUCTION:**

Secondhand smoke (SHS) in the home is a serious cause of ill-health, especially for children. SHS indoors can be indirectly measured using particulate matter monitors, and interventions have been developed using feedback from these monitors to encourage smoke-free homes. These interventions often use data that are several days out of date, as the data must be downloaded manually from monitors. It would be advantageous to access this information remotely in real-time to provide faster feedback to intervention participants.

**METHODS:**

Using off-the-shelf computer components and the Dylos DC1700 air quality monitor, a portable internet-connected monitor was developed that can send data to a server remotely. Four of these monitors were tested in homes in Israel to test the reliability of the connection. Data were downloaded from the monitor’s onboard memory and compared to the data sent to the server.

**RESULTS:**

Eight homes were monitored for 4 to 6 days, with a combined total count of 44 days. Less than 1% of data was lost, with no outage lasting longer than 1 hour 45 minutes. There was no significant difference in the mean concentrations measured in homes between mobile-transmitted data and data downloaded directly.

**CONCLUSIONS:**

This system appears to be a reliable way to monitor remotely home air quality for use in intervention studies, and could potentially have applications in other related research. Laboratories that own Dylos DC1700s may wish to consider converting them to such a system to obtain a cost-effective way of overcoming limitations in the Dylos design.

## INTRODUCTION

Secondhand smoke (SHS) in the home is a cause of indoor air pollution and a serious health risk, especially for children^1^. Interventions have been designed and trialled using air quality feedback to encourage parents not to smoke indoors^2-6^.

These interventions have generally involved the use of feedback generated after monitors have been retrieved from homes, leaving a gap between the smoking behaviour and the resulting feedback^4,7^. It may be valuable to deliver this feedback more quickly to demonstrate the positive effect on household air quality by the reduction of smoking.

Previous research by our group^5,8^ and others^9,10^ has used the Dylos DC1700 (Dylos Inc, CA, USA) successfully to monitor fine particulate matter (PM_2.5_) as a surrogate for SHS. The Dylos is a relatively inexpensive and widely used optical particle counter, considered to be well-suited for monitoring in homes. However, it has no internet capability and has numerous other limitations, including a small internal memory with a maximum data capacity of just over 6 days.

Now with the advent of the ‘internet of things’^11^, networked monitors can provide a similar function, reporting data over the internet and allowing analysis and feedback in real-time, while a monitor is still on site. However, these monitors are of variable quality and are generally dependent on a local WiFi connection rather than sending data over a mobile network^12^. A device that can use a local mobile phone signal instead can be installed more quickly, avoids the security implications of connecting to a study participant’s home network and does not rely on the presence of a fixed internet connection (important in lower socioeconomic status households in the developed world and in the global south).

It was decided to develop a prototype networked monitor based on the Dylos DC1700, using commercially available components. This monitor would send Dylos data to a website via mobile internet access, allowing real-time access and feedback on air quality in the home.

## METHODS

### System design

The Raspberry Pi computing board^13^ is a small (87.1mm × 58.5mm) inexpensive computer (currently retailing for approximately 33 GBP), useful for embedded computing, particularly where manufacturing a new device would be unnecessary, undesirable or unaffordable. While small, the computer can run sophisticated modern software in a manner similar to a PC. To allow connections to the internet, the latest model, the Raspberry Pi 3 B, comes equipped with both an Ethernet port and a WiFi chip.

As it clearly suited our needs, the Raspberry Pi was selected to form the computing portion of our monitor. For identification purposes, the combined monitoring system has been named ‘RAPID’ (a combination of the terms ‘Raspberry Pi’ and ‘Dylos’).

Although the Raspberry Pi does not include a method of accessing the internet over the mobile phone network, devices are available off the shelf that allow it to do so. This would allow for a wholly autonomous monitor that would require only electrical power to send data for as long as required. For our purposes Huawei wireless access equipment (Huawei Technologies Co. Ltd.) was selected, as it is widely available and compatible with the Raspberry Pi.

The Dylos DC1700 is a direct-reading low-cost optical particle counter, a class of instruments that detect light scattered by a laser to estimate particle size and number density in the air^14^. The Dylos estimates counts of particles larger than 0.5μm in diameter (total particle count) and number of particles larger than 2.5μm in diameter (large-particle count) per 0.01 cubic feet of air taken in^8^. In addition to recording them in its internal memory, the Dylos DC1700 reports these particle counts each minute over a 9-pin RS232 serial port in the following format:

<total particle count/100>,<large particle count/100><end of line>

These data can be converted to an estimated PM_2.5_ concentration through the use of a pre-existing equation^15^.

By design, particle-count data can be read each minute by a computer using a serial cable. The Raspberry Pi does not have a serial port, so a USB-Serial converter cable was used. Previous experience has determined that cables containing an FTDI conversion chip are most reliable, so these cables were selected for this project.

Software to record, convert, send, receive and store air quality data was developed using the Python 2.7 programming language^16^. The PythonAnywhere cloud programming service^17^ was used to host the website to receive data and store it in a database. Source code and further information on the RAPID system are available online by the authors^18^. Source code is available under an open source and free software licence.

### Monitor production

Four RAPIDs were produced using four Dylos DC1700 monitors, four Raspberry Pi 3 computers and four 16GB microSD cards. For mobile internet access, three Huawei E5330 MiFi units and one Huawei E3533 wireless dongle were used, along with SIM cards for the Partner mobile network of Israel. The total cost of the component parts to modify a Dylos DC1700 device into a RAPID is approximately 98GBP each (excluding SIM cards).

RAPIDs were produced as follows. A microSD card loaded with a Raspberry Pi-compatible operating system (Raspbian Linux) was installed in the Raspberry Pi. The custom RAPID software was then installed and set to launch as soon as the Raspberry Pi was powered on. This software was set to send data to a server running custom software designed to store the information in a database, and a unique identifier was given to the RAPID to ensure that data could be downloaded reliably later.

The Raspberry Pi was then connected to a mobile internet device and (if applicable) the WiFi network name and password were saved. The Dylos was powered on and plugged in to the Raspberry Pi using the serial-USB cable, and the Raspberry Pi was restarted to check that the completed device functioned.

### Installation in homes

The four RAPID monitors were installed in 8 homes during July and August 2017 for periods between 4 days 18 hours to 6 days 6 hours (the maximum memory capacity of the Dylos) at a time, depending on participant availability. Installation took place at variable times of the day. Monitors were installed in the main living area of the home (the living room or, in one case, a kitchen used as the main living space). Monitors were placed at least 30 cm from walls, at least 30 cm above the floor and as far as possible from windows (depending on the placement of electrical sockets in the room). Ethical approval was provided by Tel Aviv University Ethics Committee.

### Data analysis

Data from the Dylos’s internal memory and the RAPID system were compared to test the reliability of the internet connection system. Following a period of monitoring in each home, data were downloaded conventionally from the memory of each Dylos. These data were cleaned and compared to the data received and stored on the internet using the RAPID system over the same period.

Outcome variables were the arithmetic mean and median PM_2.5_ concentrations in each home using each method of data retrieval. Wilcoxon signed-rank tests were carried out to compare the mean and median PM_2.5_ concentrations recorded by each method, to determine whether significant data were lost by using the RAPID server-based data retrieval method compared with the onboard memory of the Dylos. Additionally, Spearman correlation coefficients were calculated for mean and median concentrations derived from each retrieval method. IBM SPSS Statistics software was used to conduct statistical tests^19^.

## RESULTS

### PM_2.5_ in smoking and non-smoking homes

Monitoring took place in 2 homes in Haifa, 2 in Jerusalem, 3 in Tel Aviv and one in Be’er Sheva. In four homes of smokers the people reported that they lived indoors, while for four homes there were no smokers.

Results from the homes are given in Table 1. Smoker homes had higher mean and median concentrations of PM_2.5_, but no significant difference was detected in a Mann-Whitney U test (p=0.686 in means, p=0.486 in medians). This may be due to the small sample of homes in which data was collected.

**Table 1:** Mean and median PM_2.5_ concentrations detected using the RAPID system in homes where smoking was and was not permitted, and geometric means of arithmetic mean and median PM_2.5_ concentrations

*Home ID*	*Smoking status*	*Mean PM_2.5_ (μg/m^3^ )*	*Median PM_2.5_ (μg/m^3^ )*
BE_03	Smoking permitted	82.01	13.92
HA_04	Smoking permitted	30.85	15.32
JE_03	Smoking permitted	7.10	6.05
TA_04	Smoking permitted	7.10	6.33
HA_01	Smoking not permitted	9.27	7.75
			
JE_05	Smoking not permitted	8.08	6.89
			
TA_01	Smoking not permitted	6.77	6.21
			
TA_02	Smoking not permitted	7.14	5.90
			
All smoking-permitted homes	18.90	9.51	
All smoking-not-permitted homes	7.76	6.65	

### RAPID system testing

Averaged over the eight homes, 0.57% of data was lost when using the RAPID system compared to the Dylos onboard memory — equal to 6 hours 8 minutes of data lost over 44 days of measurement. No more than 2.4% was lost in any monitoring period, while the longest single period of data loss was 1 hour 45 minutes.

**Figure 1:**
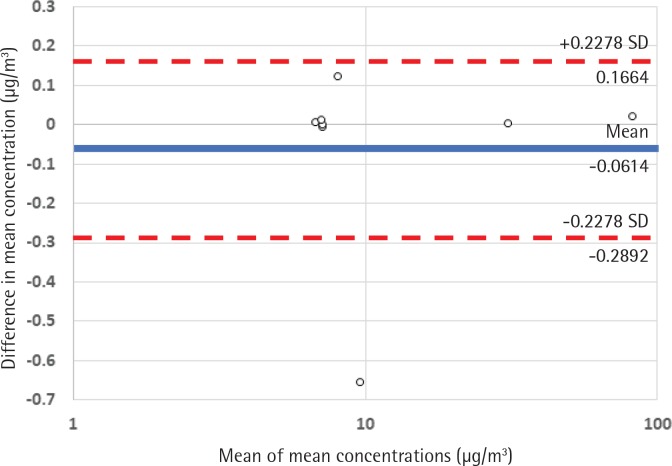
Bland-Altman plot of mean concentrations from Dylos and RAPID against the differences between each measure (n=8 ). Note the logarithmic scale used on the x-axis to account for the wide range of measurement values.

The close relationship between mean PM concentrations from the remote server and the Dylos local memory can be seen in Figure 1, a Bland-Altman plot of the differences between each measurement. Using Wilcoxon paired-rank tests no significant difference was detected between the means or medians measured using the RAPID and the onboard memory of the Dylos (p=0.327 and p=0.093, respectively). Spearman correlation coefficients showed near-perfect relationships between RAPID and Dylos data for both mean and median concentrations (ρ=1.000 and ρ=0.929, respectively).

## DISCUSSION

The results show that it is possible to use low-cost monitors combined with inexpensive, off-the-shelf consumer technology to reliably monitor PM_2.5_ in real-time within homes. This technology could be adapted with relative ease for use in smoker behaviour modification interventions.

### Advantages

Data obtained from the online server using the RAPID system were highly reliable when compared with conventional Dylos onboard memory records, demonstrating that little information was lost when being sent over the internet.

The components used to make a RAPID are widely available and inexpensive, and could therefore be accessed in many settings. The Dylos DC1700 has been widely used and research groups with existing monitors could modify their Dylos devices into RAPIDs for a lower cost than purchasing a new monitor with these features.

The core Raspberry Pi software could be customised to incorporate other monitors and sensor technology, to measure humidity and temperature.

### Disadvantages

Although errors were rare, they did occur, and could be more common in areas with poor mobile network coverage. This could be remedied by the option of connecting the RAPID to a conventional home WiFi connection.

Due to the short design period, the software used in this version of the RAPID suffered from a number of bugs that could have contributed to data loss. More recent unpublished research using these devices has resulted in substantially improved reliability.

Data loss may have occurred due to temporary failure of the mobile connection equipment or a slow internet connection (causing the RAPID software to ‘time out’ the connection with the remote server rather than remain connected indefinitely, pausing data recording). Updates made to the RAPID software following the study period now allow the system to store and re-send data that did not reach the server due to a failed internet connection.

The present form of the RAPID has used ‘off-the-shelf’ components and a number of connecting cables. The RAPID can therefore be disabled if a component is accidentally disconnected. This may not be obvious to the participant in a study due to the complexity of the device. Future work could easily remedy these problems by designing an enclosure for the Raspberry Pi and wireless access components of the device.

It should be noted that the Dylos DC1700, like all similar monitors, cannot specifically identify the source of PM_2.5_ it detects. Even with the addition of RAPID components and functionality the monitor can only be used to detect PM_2.5_ as a proxy for SHS. This could be advantageous if the equipment is to be used in other settings—for instance, for monitoring household air pollution or biomass smoke—but should be considered in the design of studies monitoring exposure to SHS. Other methods of recording information about indoor air pollution (such as diaries and data from monitoring stations on outdoor air) should be considered when using the RAPID to mitigate against this disadvantage. Additionally, it may be possible to use features of the particle-size distribution of SHS (which predominantly consists of particles 0.20–0.25 μm in diameter^20^) to detect SHS with better specificity^21^.

## CONCLUSIONS

Monitoring SHS remotely in real time is feasible without large cost. This has potential for interventions to reduce exposure to tobacco smoke and related research.
